# Efficacy and safety of probiotics in Parkinson’s constipation: A systematic review and meta-analysis

**DOI:** 10.3389/fphar.2022.1007654

**Published:** 2023-01-10

**Authors:** Li Xie, Dongmei Chen, Xinghui Zhu, Cisong Cheng

**Affiliations:** Department of Chinese Medicine, Chengdu University of Traditional Chinese Medicine, Chengdu, China

**Keywords:** probiotics, Parkinson's disease, constipation, inflammation, gut-brain axis, meta-analysis

## Abstract

**Background:** Parkinson’s disease (PD) is the most common neurodegenerative disease closely related to the immune system, among whose prodromes constipation is a representative symptom. Recent Randomized Controlled Trials (RCTs) have proved that probiotics can be used to effectively treat PD constipation, but the results are inconsistent. We performed a meta-analysis to assess the efficacy and safety of probiotic therapy on Parkinson’s constipation.

**Methods:** Questions about the research focus were constructed based on the *Participants, Intervention, Comparison and Outcomes (PICO) Criteria*. We searched electronic databases such as PubMed, Web of Science, EMBASE, Scopus, EBSCO, Cochrane and Google Scholar until March 2022 for eligible literatures. Our primary endpoints were stool frequency, stool consistency, the number of laxatives uses, UPDRS-III scores and adverse events.

**Results:** 12 eligible studies (n = 818 patients) met the inclusion and endpoint criteria. Meta-analysis results showed that constipation symptoms were improved after probiotic treatment, including an increased stool frequency (WMD = 0.94, 95% CI:0.53 to 1.34; OR = 3.22, 95% CI:1.97–5.29), an improved stool consistency (WMD = 1.46, 95% CI:0.54–2.37), a reduced use of laxatives (WMD = −0.72, 95%CI: −1.04 to−0.41), and also a reduced Parkinson’s UPDRS-III score (WMD = −6.58, 95%CI: −12.02 to −1.14); there was no significant difference in total adverse events (OR = 0.82, 95%CI:0.39–1.72).

**Conclusion:** Our analysis suggests that probiotics can be used to improve the constipation and motor symptoms for patients with Parkinson’s constipation, possibly by reducing the inflammatory response and improving gut-brain axis neuron function, whose safety also proved to be good.

## 1 Introduction

Parkinson’s disease (PD), which affects more than 1% of the elderly population in the world, is one of the most common neurodegenerative diseases (Nussbaum and Ellis, 2003). Conservative estimates indicate that the number of patients with PD will increase to 12 million worldwide by 2050 (Rocca, 2018). In addition to typical motor symptoms (MSs), PD is often accompanied by non-motor symptoms (NMSs). Many studies have demonstrated that not only the whole course of PD is accompanied by various NMSs, but they even appear before the MSs. This stage is characterized by NMSs, which lacks obvious signs of PD and is known as the prodromal stage of PD (Chaudhuri et al., 2006; Palma and Kaufmann, 2014).

Constipation is the most common prodromal symptom of PD and a predictor of PD onset (Stirpe et al., 2016), which seriously affects patients’ physical and mental health, generating a huge economic burden (Peery et al., 2019). Furthermore, patients with chronic constipation have a higher risk of developing PD (Abbott et al., 2001; Ueki and Otsuka, 2004). Constipation complicates the management of patients with PD, who often seek treatment in gastroenterology departments for reduced bowel movements and changes in their stool characteristics (Barboza et al., 2015; Pedrosa Carrasco et al., 2018).

Unfortunately, there is no effective neuroprotective or disease treatment to stop the progression of PD (Han et al., 2022). Conventional treatments for constipation are also often ineffective for Parkinsonian patients. The pathogenesis of constipation is also intermingled with PD. Measures to improve one of the symptoms of Parkinson’s constipation alone may exacerbate others (Coggrave et al., 2014). For example, anticholinergic drugs used to control PD symptoms can even make constipation worsened (Rodríguez-Ramallo et al., 2021), Constipation can also interfere with the absorption of anti-Parkinson’s drugs such as levodopa (Han et al., 2022). Therefore, it is crucial to identify a new intervention effective for preventing the progression of PD while relieving constipation from the perspective of the prodromal symptoms and risk factors of PD.

The mechanism of constipation in PD mainly involves intestinal neurotransmitter alterations (Wakabayashi et al., 1988; Anderson et al., 2007), intestinal barrier function (Forsyth et al., 2011) and gut microbes (Romano et al., 2021). Gut microbiota modulates the gut-brain axis interaction through the immune system, which is strongly associated with gastrointestinal symptoms that precede MSs, and is consistent with the hypothesis that PD pathology spreads from the gut to the brain (Mulak and Bonaz, 2015; Minato et al., 2017). It has been found in studies that probiotics alleviate constipation symptoms for patients with PD by regulating intestinal microecology (Gershon, 1981; Mulak and Bonaz, 2015). Therefore, the regulation of intestinal microbiota *via* probiotic supplementation may be a reliable treatment for Parkinson’s constipation. Therefore, to demonstrate the efficacy of probiotics in Parkinson’s constipation, this systematic review and meta-analysis need to be performed.

## 2 Methods

### 2.1 Search strategies

This meta-analysis conforms to the PRISMA guidelines (Radua, 2021). A variety of databases were used to search published studies, including PubMed, Web of Science, Scopus, Embase, EBSCO, Cochrane and Google Scholar. The last search was performed on 2 March 2022 without being limited by language. The following terms were used to search the studies: “constipation,” “functional constipation,” “dyschezia,” “colonic inertia,” “astriction,” “obstipation,” “coprostasis,” “Parkinson disease,” “Parkinson,” “secondary Parkinson’s disease,” “symptomatic Parkinson’s disease,” “Parkinsonism, symptomatic,” “secondary Parkinsonism,” “secondary vascular Parkinson’s disease,” “atherosclerotic Parkinsonism,” “probiotics,” “probiotic,” “prebiotic” and “prebiotics”. All the titles and abstracts obtained according to the search strategies were independently evaluated by two researchers to screen the studies with those that met the inclusion criteria.

### 2.2 Study selection

Studies in line with the following inclusion criteria were enrolled for this research: 1) Participants: clinical studies on patients with a confirmed PD constipation; 2) Intervention: any kind of probiotics can be included, no restrictions on different strains/doses/treatment regimens or the form of medicine, such as tablets, powders, oil suspensions or capsules; 3) Results: indicators related to constipation and Parkinson’s were accurately reported, including stool frequency, stool consistency, the number of laxative uses and UPDRS-III scores. Studies matched to the next criteria were excluded: 1) Combined use of other drugs; 2) Studies on patients with constipation triggered by surgeries, other diseases or medications; 3) Case reports, reviews, clinical experience or trial and review articles; 4) Non-human clinical studies; 5) Similar and repeated studies. Among eligible studies, the definition of the number of stools in this study was: the number of bowel movements per week, allowing the use of laxatives; the Bristol Stool Scale was used as an evaluation standard of stool consistency, with lower scores indicating harder stools.

### 2.3 Data extraction

Two researchers (X.Li and C. Dongmei) extracted the following information separately: data characteristics extracted included study characteristics (first authors, the year of publication and investigation, design, blinding and sample size); participant characteristics (age, sex and proportion of male patients, constipation diagnostic criteria and the duration of disease); intervention characteristics (types of probiotics, the mode of administration and the duration of treatment) and clinical outcomes (stool frequency, stool consistency, the number of laxative uses, UPDRS-III scores and adverse events).

### 2.4 Data analysis

This meta-analysis of data was done using RevMan 5.4.1 software. The X^2^ test was used to assess statistical heterogeneity, and the degree of heterogeneity was assessed using the I^2^ statistic. If I^2^ ≥ 50%, it indicated a high heterogeneity. We used a random-effect model to evaluate variables. If I^2^ < 50%, we used a fixed-effect model for analysis. We used Cochrane risk-of-bias tool to assess the quality of Randomized Controlled Trials (RCTs), and minors were used to assess that of non-RCTs. Sensitivity analysis studies were performed by sequentially excluding potentially-biased studies, and subgroup analyses were performed for probiotic species. Publication biases were assessed using funnel plots.

## 3 Results

### 3.1 Included studies

**FIGURE 1:**
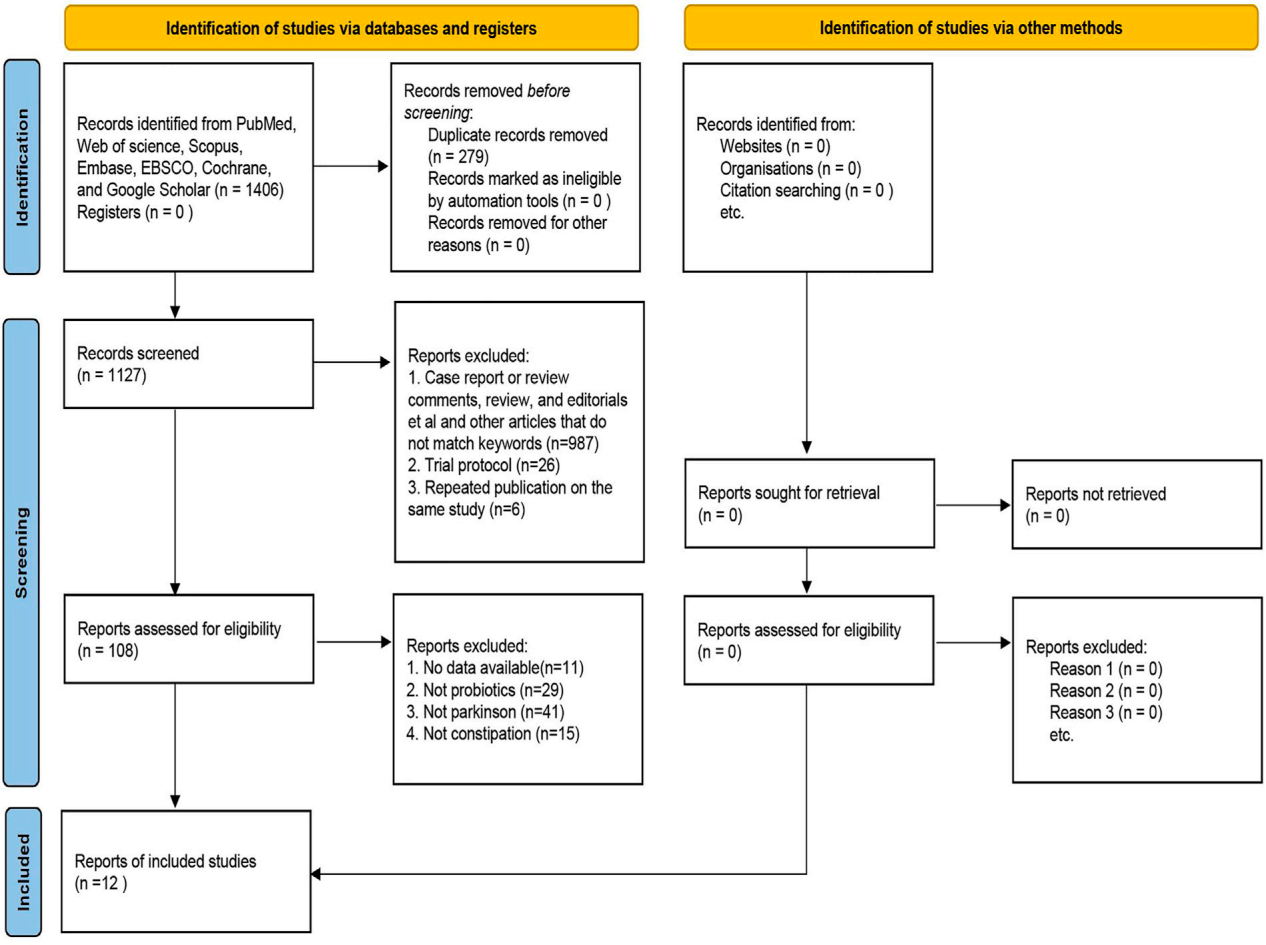
Flow chart of literature retrieval and extraction.

Figure 1 illustrates the PRISMA-compliant flowchart, describing the process of inclusion and reasons for the exclusion of references. Initially 1,406 references were searched in the database. After eliminating duplicate references, there were 1,127 references left to be screened by reading the titles and abstracts, of which, 987 references were eliminated because they failed to meet the requirements for inclusion. 108 references were finally identified for a full-text evaluation, 12 were included in the meta-analysis because they met the study eligibility criteria. Finally, nine randomized controlled trials and three single-arm clinical trials were included for meta-analysis (Cassani et al., 2011; Mazza et al., 2015; Cereda et al., 2016; Miliukhina et al., 2017; Xu et al., 2018; Ibrahim et al., 2020; Li and Sun, 2020; Sun, 2020; Wang et al., 2020; Wang-Shaoying et al., 2020; Tan et al., 2021; Yan et al., 2022), including a total of 818 patients published from 2011 to 2022. The studies were carried out in Italy, Malaysia, France, Russia, China and other countries. The types of probiotics or prebiotics used included Lactobacillus, Bifidobacterium, Bacillus and FOS; probiotics were administered in the form of yogurt, tablets, powder and capsules, with the duration of treatment ranging from 2 weeks to 16 weeks. More details are in Table 1.

**TABLE 1:** Basic characteristics of the included studies in the meta-analysis.

Study ID	Design, blinding	N of total patients	Age (mean ± SD)	*P of Males (%)*	Disease duration (mean ± SD)	Constipation definition	Probiotics	Type of probiotics/Prebiotics	Form	Treatment duration (weeks)	Loc	Adverse events		Outcomes
												Probiotic	Control	
Cassani.E.2011	Single-arm clinial trial	40	71.90 ± 6.90	90	9.75 ± 6.30	Rome Ⅲ criteria for functional constipation	Lactobacilus casei Shirota	Lactobacillus	Fermented milk	5	Italy	N/A	N/A	①②
Mazza.S.2015	Single-arm clinial trial	20	NR	60	NR	Rome Ⅱ criteria for functional constipation	Probiotic osmotic fructooligosaccharides	FOS	powder	6	France	N/A	N/A	①③
Cereda.E.2016	Randomized, double-blind, placebo-controlled clinical trial	120	71.03 ± 8.68	54.17	10.47 ± 6.57	Rome Ⅲ criteria for functional constipation	Streptococcus salivarius subsp thermophilus, Enterococ_x005f_x0002_cusfaecium, Lactobacillus rhamnosus GG, Lactobacillus acidophilus, Lactobacillus planta rum, Lactobacillus paracasei, Lactobacillus delbrueckii subsp bulgaricus, and Bifidobacterium (breve and animalis subsp lactis).	Lactobacillus, Bifidobacterium, Bacillus	Fermented milk	4	Italy	1/80	1/40	①②③⑤
Xu.L.2018	Randomized, placebo-controlled clinical trial	114	71.02 ± 11.07	55.27	1.27 ± 0.83	NR	Bifidobacterium, Lactobacillus, acidophilus, Streptococcusfaecalis	Lactobacillus, Bifidobacterium, Bacillus	tablet	8	China	1/57	2/57	②④⑤
Miliukhina.I.2017	Single-arm clinial trial	30	62.50 ± 8.50	40	8.30 ± 5.90	Rome Ⅲ criteria for functional constipation	Enterococcus (E.)	Bacillus	NR	2	Russia	N/A	N/A	①
Sun.H.R.2020	Placebo-controlled clinical trial	80	69.44 ± 6.18	58.75	2.18 ± 1.04	Rome Ⅲ criteria for functional constipation	Bifidobacterium, Lactobacillus, streptococcusthermophilus	Lactobacillus, Bifidobacterium, Bacillus	tablet	4	China	2/40	2/40	①②⑤
Li.F.2020	Randomized placebo-controlled clinical trial	40	68.50 ± 6.23	57.5	NR	Rome Ⅲ criteria for functional constipation	Bifidobacterium lactis	Bifidobacterium	powder	12	China	N/A	N/A	①
Wang.Y.Z.2020	Randomized, placebo-controlled clinicalt rial	60	66.19 ± 5.48	58.33	NR	NR	Bifidobacterium infantile, Lactobacillus acidophilus, *Enterococcus faecalis*, Bacilluscereus	Lactobacillus, Bifidobacterium, Bacillus	tablet	2	China	N/A	N/A	①
Ibrahim.A.2020	Randomized, double-blind, placebo- controlled clinical trial	53	69.78 ± 11.58	68.75	6.27 ± 1.73	Rome Ⅲ criteria for functional constipation	Lactobacillus acidophilus	Lactobacillus	Fermented milk	8	Malaysia	4/27	N/A	①④⑤
Wang.S.Y.2020	Randomized, placebo-controlled clinical trial	39	63.61 ± 6.53	51.28	7.33 ± 1.82	NR	Bifidobacteriumlongum, Lactobacillusbulgaricusand, Streptococcusthermophilus	Lactobacillus, Bifidobacterium, Bacillus	tablet	16	China	2/20	2/19	④⑤
Tan.A.H.2021	Randomized, double-blind, placebo-controlled clinical trial	72	69.22 ± 6.63	66.67	9.91 ± 6.50	Rome Ⅳ criteria for functional constipation	Lactobacillus acidophilus, Lactobacillus reuteri, Lactobacillus gasseri, Lactobacillus rhamnosus, Bifidobacterium bifidum, Bifidobacterium longum, *Enterococcus faecalis*, Enterococcus faecium	Lactobacillus, Bifidobacterium	capsule	4	Malaysia	1/34	N/A	①③⑤
Yan.T.2022	Randomized, placebo-controlled clinical trial	150	NR	56.67	NR	NR	Bifidobacterium tablets	Bifidobacterium	tablet	2	China	2/50	8/50	⑤

N/A, not applicable; NR, not reported; ①, Stool frequency; ②, Stool consistency; ③, Number of laxative use; ④, UPDRS-III scores; ⑤, Adverse events.

### 3.2 Quality assessment

**FIGURE 2:**
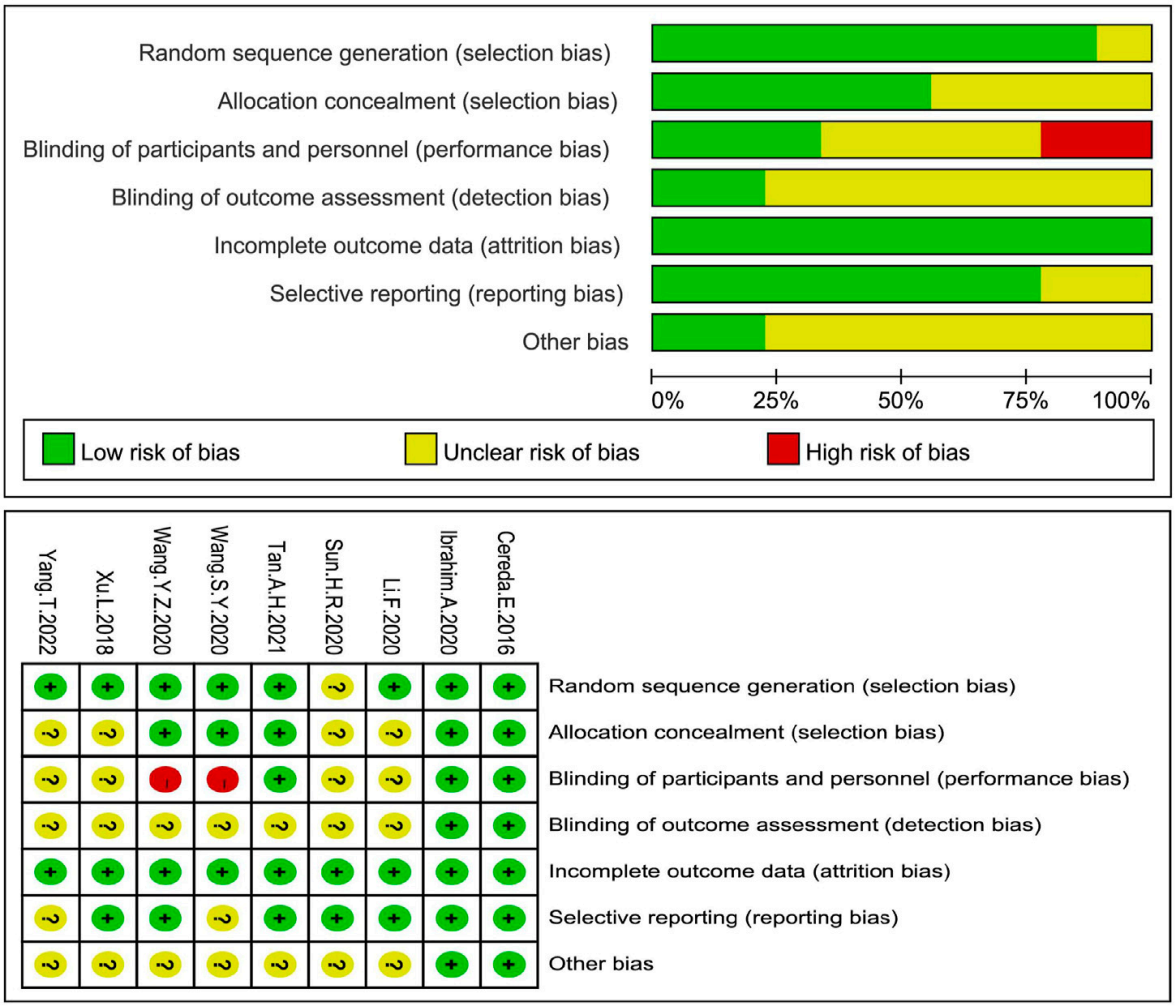
Evaluation of the risk of bias biases in all RCTs. RCTs in all groups were at the risk of biases, a low risk of biases (+), a high risk of biases (-) or an unknown risk of biases (?).

All RCTs included a probiotic intervention group and a placebo control group. 2 studies were assessed as having a low risk of biases, five had an unclear risk of biases, and two others were assessed as having a high risk of biases because of performance biases (see Figure 2). All the three non-RCTs were assessed for their quality using Minors (a methodological indicator for non-randomized studies) with a score of 16 (see Supplementary Appendix S1).

### 3.3 Stool frequency

**FIGURE 3:**
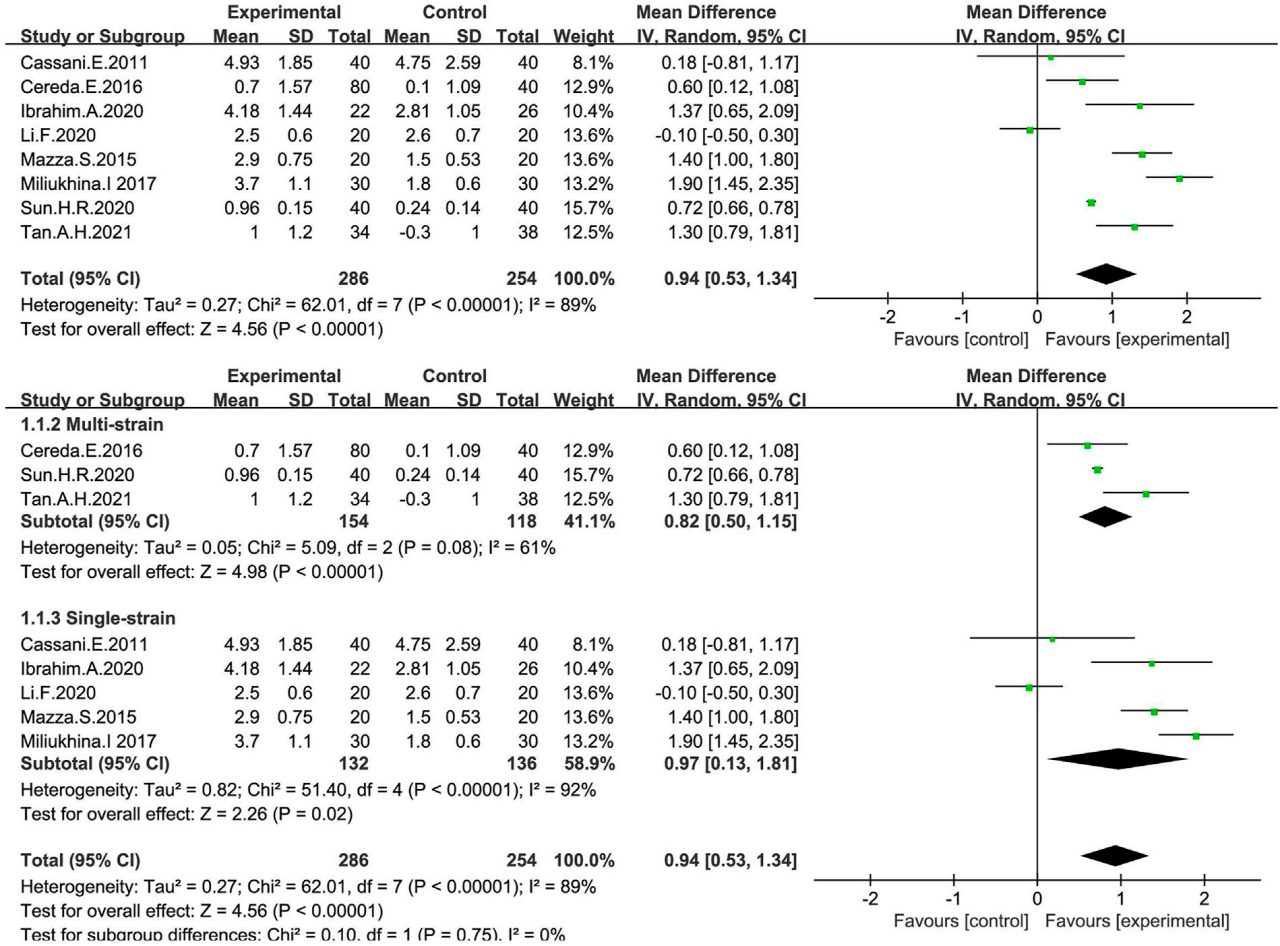
Forest plot of Stool frequency in the probiotic intervention group versus that of the control group with MD and a 95% CI analysis using a random-effects effect model.

Continuous variables were used in eight studies (Cassani et al., 2011; Mazza et al., 2015; Cereda et al., 2016; Miliukhina et al., 2017; Ibrahim et al., 2020; Li and Sun, 2020; Sun, 2020; Tan et al., 2021) to indicate the number of bowel movements per week (Figure 3). In general, the number of stools per week in the probiotic group increased by 0.94, significantly different from that in the placebo group (MD: 0.94; 95% CI: 0.53 to 1.34), but the findings were heterogeneous (I^2^ = 89%). According to different species of probiotics, we conducted a subgroup analysis, which showed that the stool frequency significantly increased with both single (MD:0.97; 95% CI:0.13–1.81) and multi strains (MD:0.82; 95% CI:0.50–1.15), especially for the single strains. However, the heterogeneity of the studies included was significant (I^2^ = 89%). By excluding each study in turn, we performed a sensitivity analysis without finding any significant change in heterogeneity, indicating the stability of the present random-effect model.

**FIGURE 4:**
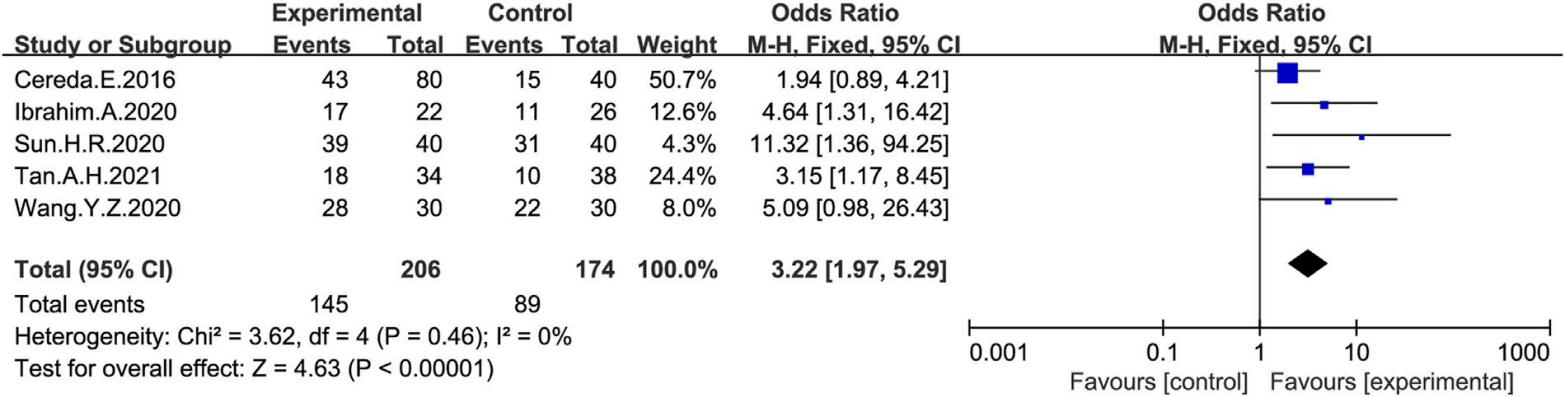
Forest plot of Stool frequency in the probiotic intervention group versus that of the control group. Fixed-effects models were used to analyze OR and 95% CI.

The overall response (treatment effective or non-responsive) to probiotic treatment was assessed in five studies (Cereda et al., 2016; Ibrahim et al., 2020; Sun, 2020; Wang et al., 2020; Tan et al., 2021) using a dichotomous approach (Figure 4), with an endpoint of achieving a mean of ≥3 BMs or an increase of ≥1 BMs per week from baseline. The meta-analysis showed that stool frequency significantly increased after a probiotic intervention, which was significant compared with the placebo group (OR:3.22; 95% CI:1.97 to 5.29; I^2^ = 0%).

### 3.4 Stool consistency

**FIGURE 5:**
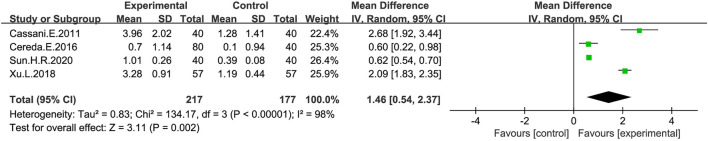
Forest plot of Stool consistency in the probiotic intervention group versus that of the control group, a random-effects model was used to analyze MD and 95% CI.

Stool consistency was measured in four studies (Cassani et al., 2011; Cereda et al., 2016; Xu et al., 2018; Sun, 2020) (Figure 5), the meta-analysis showed that stool consistency was significantly improved after a probiotic intervention, which was significant compared with the placebo group (MD: 1.46; 95% CI: 0.54–2.37), but the findings were heterogeneous (I^2^ = 98%), and we did not find significant changes in heterogeneity after a sensitivity analysis by sequentially excluding literatures, which indicated the stability of the present random-effect model.

### 3.5 The number of laxative uses

**FIGURE 6:**
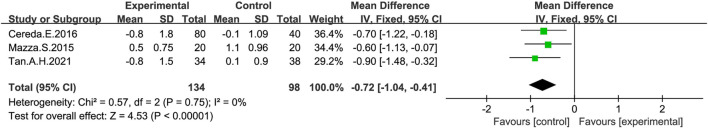
Forest plot of the Number of laxatives used in the probiotic intervention group versus that of the control group, with MD and a 95% CI analysis using a random-effects model.

Changes in the number of daily laxative uses were measured in three studies (Mazza et al., 2015; Cereda et al., 2016; Tan et al., 2021) after treatment with a probiotic intervention (Figure 6). The meta-analysis on the number of daily laxative uses in the probiotic and control group using a fixed-effect model showed a reduction in the number of daily laxative uses in the probiotic group compared to the placebo group (MD: −0.72; 95% CI: −1.04 to −0.41), with no heterogeneity in the study (I^2^ = 0%).

### 3.6 UPDRS-III scores

**FIGURE 7:**
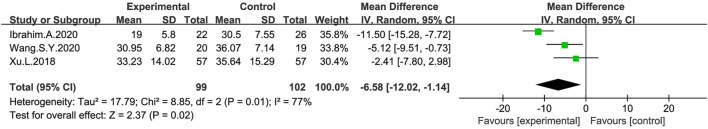
Forest plot of UPDRS-III scores in the probiotic intervention group versus that of the control group, with MD and a 95% CI analysis using a random-effects model.

UPDRS-III scores were measured after a probiotic treatment in three studies (Xu et al., 2018; Ibrahim et al., 2020; Wang-Shaoying et al., 2020) (Figure 7). The meta-analysis on the probiotic and control group using a random-effect model showed a significant reduction of UPDRS-III scores in the probiotic group compared with the placebo group (MD: −6.58; 95% CI: −12.02 to −1.14), there was heterogeneity in the studies (I^2^ = 77%), and a sensitivity analysis suggested that the Ibrahim A’s study was the source of heterogeneity, which was reduced to 0% after excluding this article.

### 3.7 Adverse events

**FIGURE 8:**
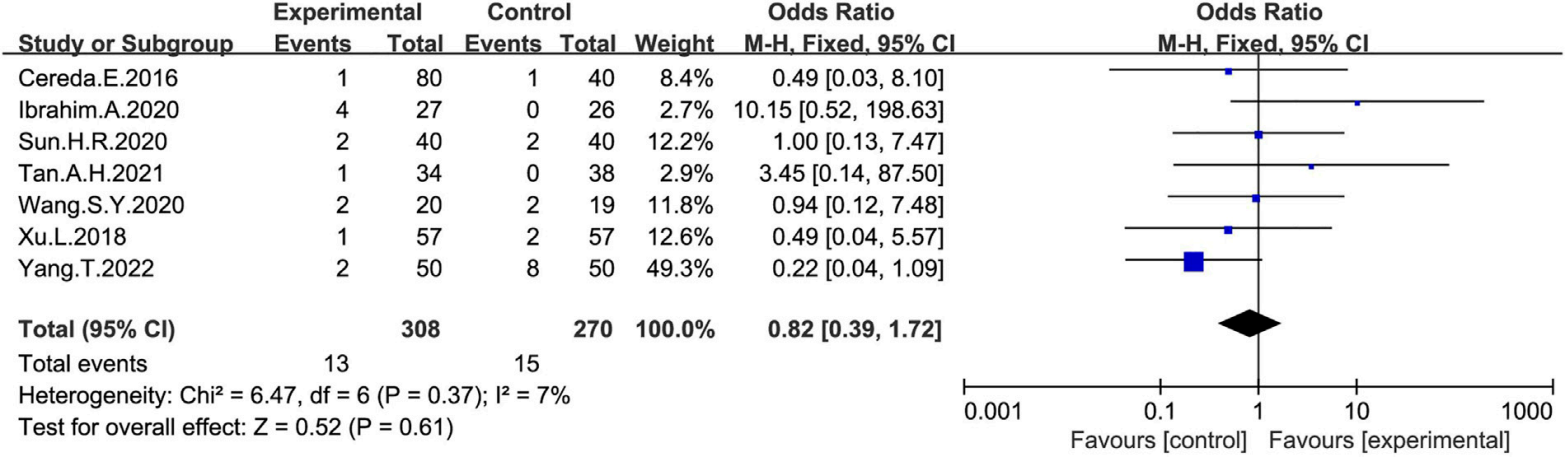
Forest plot of adverse effects in the probiotic intervention group versus that of the control group, with OR and a 95% CI analyzed analysis using a fixed-effects model.

Seven studies (Cereda et al., 2016; Xu et al., 2018; Ibrahim et al., 2020; Sun, 2020; Wang-Shaoying et al., 2020; Tan et al., 2021; Yan et al., 2022) provided information on adverse events (Figure 8), with 69% (308/448) of patients with PD constipation reporting adverse reactions after probiotic treatment, while 66% (270/410) reported adverse reactions after receiving placebo. Most of the adverse events observed in the study were abdominal distension and abdominal pains (Cereda et al., 2016; Ibrahim et al., 2020; Sun, 2020; Tan et al., 2021; Yan et al., 2022), and no serious adverse events were reported. Reduced white blood cell counts and an elevated glutamate transaminase were reported in two studies (Xu et al., 2018; Wang-Shaoying et al., 2020). However, there was no significant difference in the total number of adverse events between the probiotic and placebo group (OR:0.82; 95% CI:0.39–1.72).

### 3.8 Risk of biases

**FIGURE 9:**
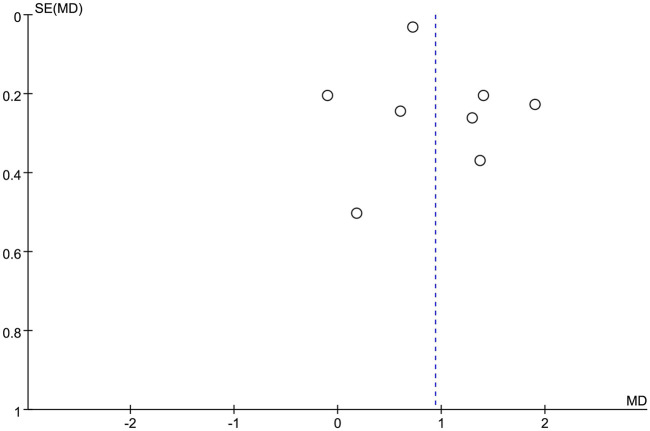
Publication bias analysis funnel plot.

Funnel plots were used to qualitatively assess publication biases, which were overall symmetrical (Figure 9). No evidence of an obvious asymmetry was shown. Therefore, the results of this meta-analysis were considered stable and reliable.

## 4 Discussion

### 4.1 Main findings and a comparison with existing literature

Our study has demonstrated that probiotics may alleviate Parkinson’s MSs while treating Parkinson’s constipation, in terms of constipation symptoms and UPDRS-III scores. This study provides evidences for the efficacy and safety of probiotic supplementation in the treatment of Parkinson’s constipation as well as a new thinking on the prevention of PD's pathological development. Stool frequency, stool consistency and the number of laxatives uses are all key variables for the evaluation of constipation treatment (Saad et al., 2010). We found that stool frequency and stool consistency increased after probiotic administration, while the number of laxatives uses decreased, which were consistent with the findings of previous studies (Huang and Hu, 2017; Zhang et al., 2020). Notably, it was found in this study that the UPDRS-III scores also decreased significantly after probiotic administration (MD: −6.58; 95% CI: −12.02 to −1.14). The UPDRS-III score is a representative variable representing the severity of MSs among patients with PD, suggesting that the motor function of patients with PD is also improved after an oral probiotic treatment. It is a novel finding relative to previous studies (Xiang et al., 2022), which indicates that through probiotic therapy, constipation symptoms can not only be relieved, but also may be effective in preventing the progression of PD.

Considering that the number of probiotic species greatly influences the therapeutic effect, we performed a subgroup analysis (see Figure 3). It was found that single strains promoted a greater increase in stool frequency compared to multiple strains (MD:0.94; 95% CI:0.53–1.34). Notably, although many studies have been focused on comparing the efficacy of multiple strains with that of a single strain, the results are quite different (Chapman et al., 2013; Wilkins and Sequoia, 2017). A study showed that in most cases, multi-strain probiotics were less effective than single-strain ones (McFarland, 2021). Stronger and more in-depth research is needed in this area.

Our study has demonstrated that probiotics may alleviate Parkinson’s MSs while treating Parkinson’s constipation, in terms of constipation symptoms and UPDRS-III scores.

### 4.2 Implications for clinical practice

**FIGURE 10:**
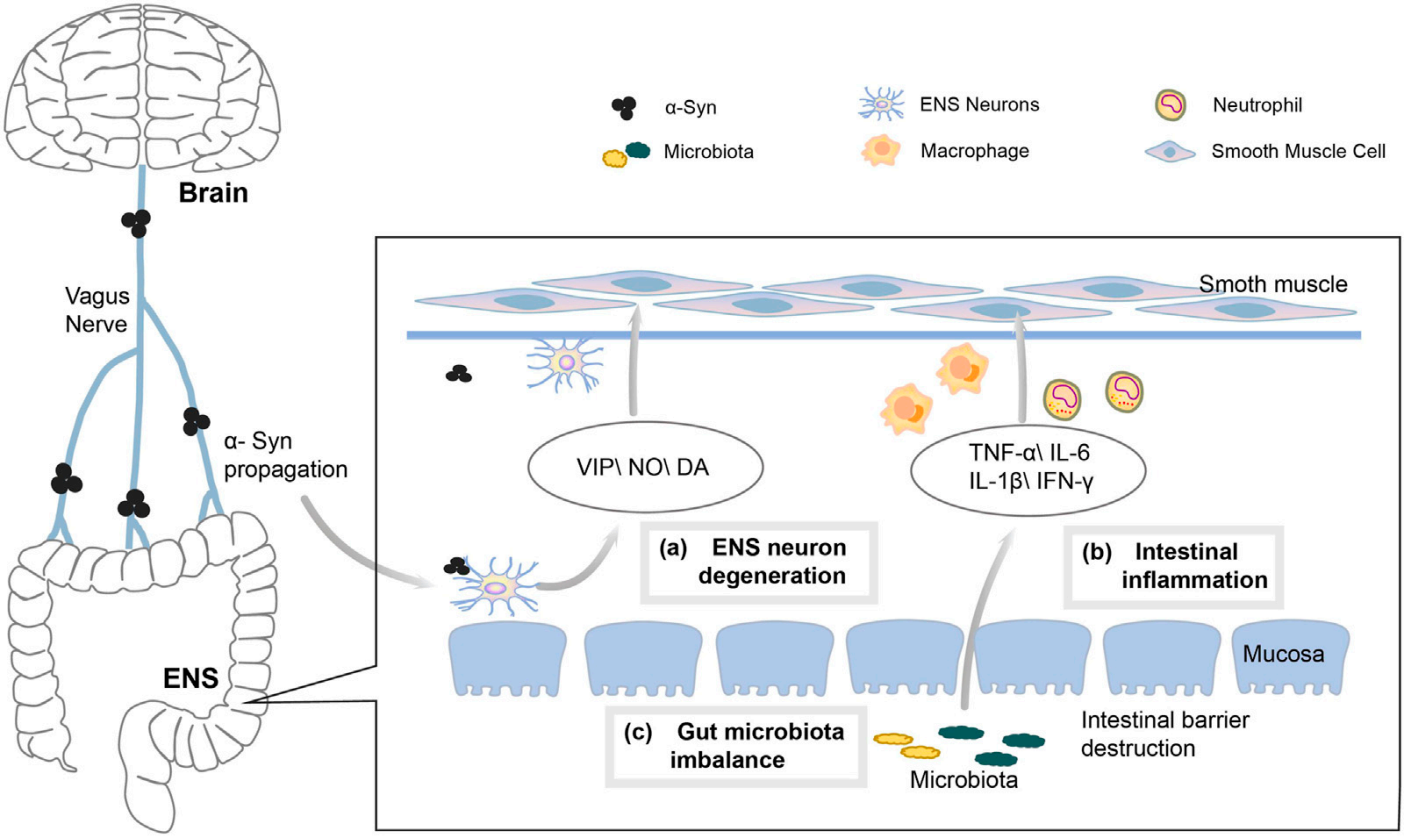
Mechanism of PD causing constipation.

A typical pathological feature of PD is the aggregation of alpha-synuclein (α-syn) within the central nervous system (CNS). As α-syn progresses, the main symptoms of PD gradually appear, including premotor symptoms, sleep and motor disturbances and cognitive as well as emotional problems (Braak et al., 2003; Fahn, 2003). However, α-syn also begins in the submucosa from the enteric nervous system (ENS), and then travels retrogradely through the gut-brain axis to the CNS, thus causing MSs of PD. Constipation is one of the most common NMSs of PD, with a prevalence of 24.6%–63% among patients with PD (Stocchi and Torti, 2017). This is mainly due to the dysfunction of colonic motility in patients with PD, and many factors may contribute to alterations in colonic transit (Figure 10), including the following factors.

#### 4.2.1 Degeneration of ENS neurons

Usually, gastrointestinal motility disorders in PD are mainly caused by ENS and vagal abnormalities (Quigley, 1996). The large number of sensory and motor neurons contained in the ENS control the contraction of the gastrointestinal tract (GIT) muscles and the movement of transmucosal fluid (Furness, 2012). The changes in intestinal neurons and neurotransmitters play an important role in the pathogenesis of PD constipation (Wakabayashi et al., 1988; Anderson et al., 2007). The number of neurons decreases with the accumulation of α-syn in ENS, which may lead to the dysregulation of intestinal fluid secretion and neurotransmitter function, thus further impeding intestinal motility and promoting constipation (Lebouvier et al., 2010). In addition, the aggregation of α-syn causes vagal atrophy, which affects the role of vagus nerves in promoting digestive enzymes, hormone secretion and smooth muscle bowel motility meanwhile also inhibiting gastrointestinal motility, leading to the development of constipation (Del Tredici and Braak, 2016).

#### 4.2.2 Inflammatory factors

PD is associated with the inflammation of GIT, and a significant increase has been found in studies on the expression of pro-inflammatory cytokines in colonic specimens from patients with PD (Devos et al., 2013). A reduced level of short-chain fatty acids (SCFAs) in the intestine of patients with PD leads to an impaired intestinal barrier and an accelerated ENS neuroinflammation (Hirayama and Ohno, 2021). At the same time, the induced inflammatory mediators promote the infiltration of neutrophils and macrophages into the smooth muscle layer as well as the production of nitric oxide (NO), resulting in a weakened contraction of intestinal smooth muscles while promoting the development of constipation (Türler et al., 2006).

#### 4.2.3 Intestinal microecological disorders

Patients with PD are prone to intestinal flora dysbiosis, Unger et al. found that the bacterial phylum Bacteroidetes and the bacterial family Prevotellaceae were reduced, meanwhile Enterobacteriaceae was more abundant in fecal samples from patients with PD (Unger et al., 2016). The dysbiosis of intestinal microecology leads to a significant decrease in the concentration of SCFAs, the products of bacterial fermentation in intestines, which affects the development of constipation by influencing the energy supply of colonocytes (Kaiko et al., 2016), the intestinal barrier (Kelly et al., 2015), the inflammatory response (Inan et al., 2000) and neuronal functions (Vicentini et al., 2021). Probiotics affect intestinal luminal pH, mucosal absorption and secretion as well as colonic motility by altering the intestinal microenvironment (Quigley and Spiller, 2016).

#### 4.2.4 Adverse drug reactions and lifestyles

Anti-PD drug use is another cause of constipation, and a significant increase has been found in studies on the incidence of constipation among patients with PD treated with levodopa, possibly due to the delayed gastric emptying triggered by levodopa (Pagano et al., 2015; Bestetti et al., 2017). Other anti-PD drugs, such as ropinirole, bromocriptine and piribedil, can promote an increased incidence of constipation (Li et al., 2017). In the later stages of disease progression, patients with PD move slowly, who are even bedridden for long periods of time, resulting in a low colon motility and symptoms including a prolonged defecation as well as difficulties in passing stools. In addition, the dehydration that older patients with PD are prone to causes the release of pressin and aldosterone, which leads to an excessive absorption of water and salt in the colons, resulting in constipation (Read et al., 1995; Thiyagalingam et al., 2021).

### 4.3 Intestinal flora affects PD constipation by regulating the brain-gut axis

**FIGURE 11:**
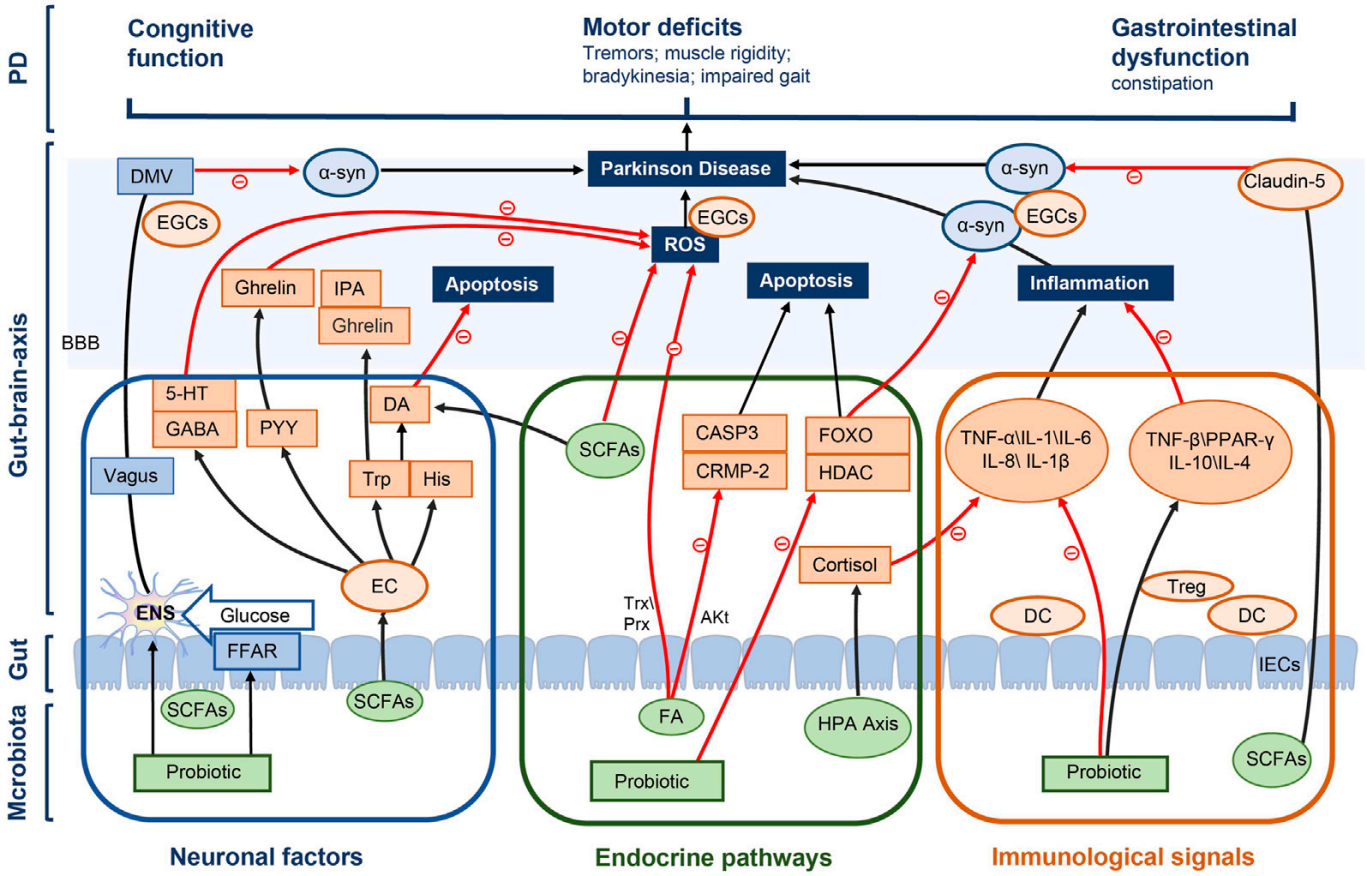
Potential mechanisms of the probiotic modulation in PD constipation.

The regulation of intestinal flora plays an important role in the development and treatment of PD constipation. Intestinal flora consists of a variety of bacteria in the GIT that live in symbiosis with the human host. Most species of gut flora belong to phyla Firmicutes, including Clostridium coccoides, Clostridium leptin and lactobacillus, as well as the Bacteroides phylum, including Bacteroides and Prevotella (Qin et al., 2010). With the development of macrogenomics, the association between alterations in gut flora and disease states is becoming clearer. Clinical studies have also shown that the number of probiotics in the stool of patients with constipation is significantly reduced. Supplementing probiotics is an effective way to relieve constipation and intestinal flora imbalance (Dimidi et al., 2017). In another randomized controlled trial, stool specimens from elderly patients were examined before and after probiotic administration, through which a significant increase in B. breve, B. longum and B. adolescentis was observed after a probiotic intervention (Kondo et al., 2013). This provides an additional rationale for treating constipation with probiotics through the modulation of intestinal microbiota. Additionally, gut microbes can influence brain functions through the gut because of the bidirectional communication in the gut-brain axis, which affects the progression of PD. The gut-brain axis consists of the entire gut microbiota, ENS, parasympathetic and sympathetic nervous systems, CNS, neuroendocrine connections, humoral pathways, cytokines, neuropeptides as well as signaling molecules (O'Mahony et al., 2015), which is a bidirectional neuroendocrine system that communicates between GIT and CNS. In the gut, which is in contact with the gut microbiota and the brain as well as isolated by the blood-brain barrier (BBB), microbiota acts on the CNS through many hormones and metabolites secreted by the intestinal epithelial cells (IECs) to achieve microbial effect on the gut-brain axis. We hypothesized that probiotics affected PD constipation through gut-brain axis, which were summarized as follows in three ways (Figure 11).

#### 4.3.1 Neuronal factors

Intestinal flora can directly stimulate electrical signals in the ENS and the dorsal motor nucleus of vagus (DMV) by propagating signals through the vagus nerves to affect the brain center (Clarke et al., 2014), thus reducing the accumulation of α-syn and alleviating the motor deficits of patients with PD, including tremors, muscle rigidity, bradykinesia and an impaired gait (Sampson et al., 2016). Various probiotics release SCFAs by digesting fibers in the gut (Macfarlane and Macfarlane, 2012; Han et al., 2022), and SCFAs affect the energy supply for CNS cell metabolism through the brain-gut axis. e.g., butyrate and propionate are transported across the BBB into the CNS *via* monocarboxylate transporters (mCTs), which are then taken up through mCTs on glial cells and neurons (Pellerin, 2005). In addition, SCFAs enable signaling through G-protein-coupled free fatty acid receptors FFAR2 and FFAR3 on the intestinal epitheliums, initiate gluconeogenesis, act on the vagus nerves and DMV, affect CNS transmission processes as well as facilitate the cognitive recovery of patients with PD (Delaere et al., 2012) (Lacassagne and Kessler, 2000; Lal et al., 2001).

The accumulation of reactive oxygen species (ROS) activates neuroinflammation in microglia, which is one of the main factors for the development of PD (Franceschi and Campisi, 2014), because the high energy demand of brain neurons leads to a high sensitivity of them to ROS-induced oxidative damage. Microbiota effectively inhibits ROS accumulation, either directly or by stimulating the production of large amounts of gamma-aminobutyricacid (GABA) through many neural metabolites in the intestinal epithelia, such as Lactobacillus and Bifidobacterium strains, meanwhile promoting 5-hydroxytryptamine (5-HT) production by regulating GABA concentration. The expression of 5-HT is also important for neurological functions of the brain, which is expressed in both the ENS and CNS. SCFAs can modulate intestinal endocrine 5-HT action in the ENS (Evans et al., 2013), increasing intestinal motility to relieve constipation and further affecting α-syn misfolding in the brain-gut axis. Most (>90%) of a body’s serotonin is produced in the EC cells of the intestines (Gershon, 2013). SCFA induces the action of EC cells to express tryptophan 6-hydroxylase 1, so as to regulate 5-HT signaling (Nankova et al., 2014).

The ability of intestinal bacteria to produce ghrelin makes it a promising therapeutic target for PD. Gut hormones such as peptide YY(PYY) and cholecystokinin, produced by EC cells under the influence of microbiota, interact with ghrelin signaling to reduce ROS accumulation, protect mitochondrial integrity and therefore promote an anti-apoptotic environment meanwhile protecting against neuronal functions (Liu et al., 2009). EC cells also produce a number of neuroactive factors, including tryptophan (Trp) and histidine (His), which are neurotransmitters and neuroactive molecules that enter the circulation and cross the BBB, affecting CNS signaling. Intestinal flora can regulate the plasma concentration of Trp, a precursor of essential amino acids and pentraxin as well as a key neurotransmitter in the ENS and CNS, further stimulating the production of gut hormones in the CNS, such as gastrin and indole-3-propionic acid (IPA), which exert neuroprotective effect on the CNS. Trp and tyrosine hydroxylase gene expression regulates the synthesis of neurotransmitters such as Dopamine (DA) and 5-HT (Nankova et al., 2014), which is also very important in the progression of PD.

#### 4.3.2 Endocrine pathways

The gut microbiota acts as a virtual endocrine organ that produces and regulates the metabolic capacity of a wide range of compounds producing a series of endocrine signaling molecules, which affect many biochemical pathways in the systems and brain, in turn affecting the functions of distal organs and systems. For example, short-chain fatty acids are produced during carbohydrate metabolism, such as butyrate and propionate, which provide an important source of nutrition and regulate host digestion. SCFAs can readily enter the circulation from the gut and are transported across the BBB *via* monocarboxylate transporters (mCTs). Butyric acid, a metabolite of intestinal flora, inhibits histone deacetylase (HDAC) activity and induces forkhead box o (FOXO) to cause cell deaths through the transcriptional regulation of apoptotic genes, and its induction of autophagy genes has been shown as a key mechanism for neurological function protection (Xu et al., 2011). In addition, Parkinson’s α-Syn aggregation is reduced in a FOXO-dependent manner with butyrate, which has a direct neuroprotective potential (Koh et al., 2012; Beharry et al., 2014).

Ferulic acid (FA) is another key class of molecules produced directly by microbiota that can effectively inhibit the production and activity of ROS and is considered as a powerful scavenger of ROS (Trombino et al., 2013), which may be achieved by regulating peroxiredoxin reductase (PRX) and thioredoxin (Trx) (Patenaude et al., 2005). PRX and TRX are ubiquitous antioxidant proteins that regulate cell proliferation and apoptosis while providing neuroprotection (Sung et al., 2014). In addition, FA also inhibits the GSK3 β pathway by upregulating Akt signaling, thus preventing the expression of CRMP-2 and Casp3, thereby inhibiting apoptosis (Gim et al., 2013). Another part of gut microbiota involved in the regulation of the brain-gut axis is the stimulation of the HPA axis, during which the release of cortisol can suppress inflammatory responses and influence brain plasticity development as well as subsequent biological systems (Westfall et al., 2017).

#### 4.3.3 Immunological pathways

A reason for the probiotic treatment of PD constipation is to reduce the degenerative loss of dopaminergic neurons by reducing the inflammatory response through the modulation of immune function. An inflammatory environment has been shown to increase α-Syn aggregation, which may further activate microglia upon contact, encouraging a feed-forward cascade that leads to more α-Syn aggregation and propagation as well as disease development. After intestinal microecological disorders, intestinal permeability increases, which induces the relevant immune response of pro-inflammatory cells. Inflammation is reduced with probiotics by promoting the balance of the intestinal microecological structure and regulating inflammatory cytokines through the MAPK and NF-kB pathway. It has been found in studies (Borzabadi et al., 2018; Perez Visñuk et al., 2020) that probiotics can be used to increase the production of anti-inflammatory factors and reduce the expression of inflammatory factors for patients with PD.

In addition, the GIT mucosal epithelial tissues contain a large number of antigen-presenting localized innate immune cells, such as macrophages and dendritic cells. Through such localization, immune cells are put in close proximity to the gut microbiota, invading pathogens and antigens that breach the protective epithelial barrier, allowing for an effective immunological communication between the external environment and the systemic immune system. Microbe-associated molecular patterns (MAMPs) on the surface of the microbiota such as lipoteichoicacid (LTA) and surface layer protein A (SlpA) directly stimulate receptors on immune cells, such as toll-like receptors (TLR) and intercellular cell adhesion molecule (ICAM) on dendritic cells (DCs). Anti-inflammatory responses are also propagated during this interaction through the upregulation of anti-inflammatory factors (IL-10 and IL-4) (Lavasani et al., 2010), which also inhibits pro-inflammatory cytokines (TNF-α, IL-1β and IL-6). In addition, some microbiota also directly inhibits pro-inflammatory cytokines, such as the effect of Bacillus animalis on IL-6. Histamine is a biogenic monoamine released from Lactobacillus, which can be involved in the regulation of immune cells meanwhile acting as a neurotransmitter in the brain. Histamine induces anti-inflammatory responses through its receptor H4R by activating several signaling factors including JAK-STAT, MAPK/ERK and PI3K, ultimately leading to the release of anti-inflammatory cytokines, the regulation of dendritic cell functions and the recruitment of T regulatory cells to the sites of acute inflammation (Schneider et al., 2002; Morgan et al., 2007).

In addition, with the probiotic production of SCFAs, the expression of occludin and Claudin-5 can be regulated, which can protect the BBB. Changes in the intestinal flora lead to alterations in barrier immune function meanwhile affecting the nervous systems including the ENS and EGCS. Inflammatory responses trigger microglia maturation, affecting ENS functions, the intestinal motility and α-synaptic nuclear protein misfolding (Forsyth et al., 2011; De Vadder et al., 2014). Probiotics can be used to further delay the pathological progression of PD by strengthening the intestinal barrier function, reducing immune and inflammatory responses as well as improving ENS functions by regulating EGCS homeostasis and affecting α-synaptic nuclear protein misfolding.

## 5 Limitations

We used a rigorous approach to reduce publication biases in this meta-analysis. Two investigators performed literature inclusion and data extraction separately, and where a significant heterogeneity was revealed through data analysis, we used a random-effect model to reduce the possibility of overestimating the treatment effect. In addition, subgroup analysis was used for the number of probiotic species to assess treatment effect; data on adverse events was extracted to supplement probiotic safety in PD constipation, and funnel plots proved to be overall symmetric.

However, this study has some limitations. First of all, most of each study included was conducted in different countries; therefore, different genetic constitutions as well as cultural and environmental factors (eating habits, etc.) might have contributed to the heterogeneity of the studies. Secondly, the heterogeneity of some of the meta-analyses was significant, implying differences in those studies. This might be due to the different types and doses of probiotics in the studies, as well as other factors, such as physical condition and eating habits, etc. Thirdly, the number of existing studies on RCTs is limited, and the sample size of some studies is small, which affect the rigorness of the research results to a certain extent. However, due to the diversity of probiotic species and strain specificity, the efficacy and effect of various types of probiotics on constipation may be inconsistent, and further studies on the effect of different strains, doses as well as treatment durations of probiotics on constipation are needed.

For subsequent studies, we have the following suggestions. First of all, bigger sample sizes and more adequate pilot studies using standardized measurements should be accumulated to obtain fuller evidences. Secondly, more subgroup studies should be added, such as different strains and doses of probiotics.

## 6 Conclusion

It is found in this meta-analysis that probiotics can not only be used to treat Parkinson’s constipation symptoms, but also alleviate the MSs for patients with PD. Probiotics influence the pathological development of PD by modulating brain-gut axis functions and immune responses. However, the current randomized controlled trial study still has some limitations, thus further RCTs are warranted.

## Data Availability

The original contributions presented in the study are included in the article/Supplementary Material, further inquiries can be directed to the corresponding author.
