# Supplementary material for: Efficacy and safety of probiotics in Parkinson’s constipation: A systematic review and meta-analysis
Source: Front Pharmacol. 2023 Jan 10;13:1007654. doi: 10.3389/fphar.2022.1007654 (PMC9871263; doi:10.3389/fphar.2022.1007654)
Supplement: Supplementary file 2 [file Table3.DOCX]

| **Appendix 1: Search Strategy** | | |
| --- | --- | --- |
| **Database** | # | **Search syntax** |
| **1)Pubmed** | 1 | “Constipation”[Mesh] |
|  | 2 | “Functional constipation”[Title/Abstract] OR “Dyschezia”[Title/Abstract] OR “Colonic inertia” [Title/Abstract]OR “Astriction” [Title/Abstract]OR “Obstipation”[Title/Abstract] OR “coprostasis”[Title/Abstract] |
|  | 3 | #1 OR #2 |
|  | 4 | “Parkinson disease”[Mesh] |
|  | 5 | “Parkinson” [Title/Abstract]OR “Secondary Parkinson Disease”[Title/Abstract] OR “Symptomatic Parkinson Disease”[Title/Abstract] OR “Parkinsonism, Symptomatic”[Title/Abstract] OR “Secondary Parkinsonism”[Title/Abstract] OR “Secondary Vascular Parkinson Disease”[Title/Abstract] OR “Atherosclerotic Parkinsonism”[Title/Abstract] |
|  | 6 | #4 OR #5 |
|  | 7 | “Probiotics”[Mesh] |
|  | 8 | “Probiotic” [Title/Abstract]OR “Prebiotic” [Title/Abstract] OR “Prebiotics”[Title/Abstract] |
|  | 9 | #7 OR #8 |
|  | 10 | #3 AND #6 AND #9 |
| **2)Embase** | 1 | “Constipation”/exp |
|  | 2 | (“Functional constipation” OR Dyschezia OR “Colonic inertia” OR Astriction OR Obstipation OR coprostasis):ti,ab,de |
|  | 3 | “Parkinson disease”/exp |
|  | 4 | (Parkinson OR “Secondary Parkinson Disease” OR “Symptomatic Parkinson Disease” OR “Parkinsonism, Symptomatic” OR “Secondary Parkinsonism” OR “Secondary Vascular Parkinson Disease” OR “Atherosclerotic Parkinsonism”):ti,ab,de |
|  | 5 | “Probiotics”/exp |
|  | 6 | (Probiotic OR Prebiotic OR Prebiotics):ti,ab,de |
|  | 7 | (#1 OR #2) AND (#3 OR #4) AND (#5 OR #6) |
| **3)Cochrane CENTRAL** | 1 | MeSH descriptor:[Constipation] this term only |
|  | 2 | (“Functional constipation” OR Dyschezia OR “Colonic inertia” OR Astriction OR Obstipation OR coprostasis):ti,ab,kw |
|  | 3 | MeSH descriptor:[ Parkinson disease] this term only |
|  | 4 | (Parkinson OR “Secondary Parkinson Disease” OR “Symptomatic Parkinson Disease” OR “Parkinsonism, Symptomatic” OR “Secondary Parkinsonism” OR “Secondary Vascular Parkinson Disease” OR “Atherosclerotic Parkinsonism”):ti,ab,kw |
|  | 5 | MeSH descriptor:[Probiotics] this term only |
|  | 6 | (Probiotic OR Prebiotic OR Prebiotics):ti,ab,kw |
|  | 7 | (#1 OR #2) and (#3 OR #4) and (#5 OR #6) |
| **4) Scopus** | 1 | TITLE-ABS-KEY(Constipation OR “Functional constipation” OR Dyschezia OR “Colonic inertia” OR Astriction OR Obstipation OR coprostasis) |
|  | 2 | TITLE-ABS-KEY("Parkinson disease" OR Parkinson OR “Secondary Parkinson Disease” OR “Symptomatic Parkinson Disease” OR “Parkinsonism, Symptomatic” OR “Secondary Parkinsonism” OR “Secondary Vascular Parkinson Disease” OR “Atherosclerotic Parkinsonism”) |
|  | 3 | TITLE-ABS-KEY(Probiotics OR Probiotic OR Prebiotic OR Prebiotics ) |
|  | 4 | #1 AND #2 AND #3 |
| **5)Web of science** | 1 | TS=(Constipation OR “Functional constipation” OR Dyschezia OR “Colonic inertia” OR Astriction OR Obstipation OR coprostasis) |
|  | 2 | TS=("Parkinson disease" OR Parkinson OR “Secondary Parkinson Disease” OR “Symptomatic Parkinson Disease” OR “Parkinsonism, Symptomatic” OR “Secondary Parkinsonism” OR “Secondary Vascular Parkinson Disease” OR “Atherosclerotic Parkinsonism”) |
|  | 3 | TS=(Probiotics OR Probiotic OR Prebiotic OR Prebiotics ) |
|  | 4 | #1 AND #2 AND #3 |
| **6)EBSCO** | 1 | TX=(Constipation OR “Functional constipation” OR Dyschezia OR “Colonic inertia” OR Astriction OR Obstipation OR coprostasis) |
|  | 2 | TX=("Parkinson disease" OR Parkinson OR “Secondary Parkinson Disease” OR “Symptomatic Parkinson Disease” OR “Parkinsonism, Symptomatic” OR “Secondary Parkinsonism” OR “Secondary Vascular Parkinson Disease” OR “Atherosclerotic Parkinsonism”) |
|  | 3 | TX=(Probiotics OR Probiotic OR Prebiotic OR Prebiotics ) |
|  | 4 | #1 AND #2 AND #3 |
| **7)Google Scholar** | 1 | intitle:(Constipation OR “Functional constipation” OR Dyschezia OR “Colonic inertia” OR Astriction OR Obstipation OR coprostasis)AND ("Parkinson disease" OR Parkinson OR “Secondary Parkinson Disease” OR “Symptomatic Parkinson Disease” OR “Parkinsonism, Symptomatic” OR “Secondary Parkinsonism” OR “Secondary Vascular Parkinson Disease” OR “Atherosclerotic Parkinsonism”) AND (Probiotics OR Probiotic OR Prebiotic OR Prebiotics ) |
